# Development of in-house software to process real-time cine magnetic resonance images acquired during 1.5 T MR-guided radiation therapy

**DOI:** 10.1038/s41598-025-15107-4

**Published:** 2025-08-12

**Authors:** Jiwon Sung, Yeonho Choi, Jina Kim, Jun Won Kim, Jihun Kim

**Affiliations:** Department of Radiation Oncology, Gangnam Severance Hospital, Yonsei University College of Medicine, Seoul, South Korea

**Keywords:** MR-linac, 2D cine MR image, MR guided radiotherapy, MATLAB, Radiotherapy, Biomedical engineering

## Abstract

This study aimed to develop and publicly release an in-house software package that converts the binary file format of 2D cine magnetic resonance (MR) images acquired through the Treatment Session Manager (TSM) of MOSAIQ on the Elekta Unity (Elekta AB, Stockholm, Sweden) into standard readable data formats. The software was developed using MATLAB (MathWorks, Natick, MA, USA) and includes an automatic image-sorting algorithm to classify the images into coronal, sagittal, and axial planes. To verify the geometric accuracy of the converted images, they were acquired from an MRgRT motion management QA phantom, both with and without motion. For the converted images without motion, the geometric size of the phantom was measured and compared with the known values. For the images acquired with motion, the magnitudes measured using the converted cine MR images were compared with the artificially introduced motions. The results showed that the 2D cine MR images in the binary file format were successfully converted into the metadata/DICOM format and accurately classified into different planes. The accuracies of the geometric size and motion in the converted 2D cine MR images were greater than 99.6% and 94.2%, respectively. This software is expected to be useful for 1.5 T MR-linac users in analyzing internal organ motion during radiation treatment.

**Supplementary Information:**

The online version contains supplementary material available at 10.1038/s41598-025-15107-4.

## Introduction

The technological combination of magnetic resonance (MR) imaging and a linear accelerator (linac), referred to as an MR-linac, has opened a new era of MR-guided radiation therapy (MRgRT)^1–3^. Whereas cone-beam CT is widely used for daily treatment imaging, MR can provide a higher geometric resolution of soft tissues on a daily basis^4–6^. With the technological advantage of monitoring daily patient geometry, the original radiation treatment plan can be adapted, and MR-guided adaptive radiation therapy may have the potential to improve treatment quality^7,8^.

Radiotherapy has several strengths that can be enhanced through the use of an MR-linac. In conventional linac radiotherapy, a challenge in treating patients is the proximity of tumors to dose-limiting organs at risk (OARs), which can limit long-term tumor control. For example, radiotherapy for solid tumors such as pancreatic adenocarcinoma has historically been very challenging because of the proximity of the small bowel, stomach, and colon, which limits the radiation doses that can be delivered to the tumor^9^. Adaptive radiotherapy using an MR-linac has the potential to overcome these limitations^10,11^.

Another outstanding advantage is the acquisition of two-dimensional (2D) cine MR images during radiation treatment, which enables real-time tracking of target motion. ViewRay MRIdian (ViewRay Inc., Oakwood, USA), an MR-linac, automatically controls the treatment beam that irradiates the target when it is at the desired position, using real-time 0.35 T MR images acquired during treatment to track the motion of the target^12,13^. Elekta Unity (Elekta AB, Stockholm, Sweden) is another MR-linac that utilizes high-resolution 1.5 T MR imaging to enable real-time monitoring of patient organ motion during treatment, making it useful for research related to organ motion and functional MRIs acquired during treatment^14–16^. Recently, Elekta Unity is also capable of automatic gating treatment through Comprehensive Motion Management (CMM), which recevied Food and Drug Administration (FDA) in 2023^17^ and is now being increasingly adopted in clinical practice.

Although 2D cine MR images are useful not only for automatic gating treatment systems but also for analyzing organ motion during the treatment, they are stored in an unknown data format that is not compatible with commonly used commercial software. This makes it difficult for clinical staff and researchers to directly analyze or use the data for research purposes.

Akdag et al. acquired 2D cine MR images during stereotactic arrhythmia radioablation using a 1.5 T MR-linac and analyzed real-time cardiorespiratory motion using in-house developed C + + software^18^. Kim et al. developed in-house software to process cine MR images acquired from an MR simulator rather than an MR-linac^19^. Jassar et al. published the results of applying image registrations to cine MR images acquired during treatment using the motion management research package (MMRP) provided by Elekta^20^. As of the publication of this article, there is no publicly or commercially available software that supports the processing of cine MR images acquired with a 1.5 T MR-linac. Cine MR images generated by the Elekta Unity are stored in an unknown binary format, limiting their use for clinical and research purposes at many institutions.

Therefore, we aimed to develop a software tool capable of converting 2D cine MR images saved in a nonstandard binary format into a commonly used metadata (MHA) file and Digital Imaging and Communications in Medicine (DICOM) format for medical imaging in this study. Additionally, the accuracy of the converted cine MR images in depicting the geometry and movement of an imaged object was evaluated. To this end, we acquired 2D cine images of a motion management quality assurance (QA) phantom with and without motion and compared the geometry and motion magnitude in the converted cine MR images to the ground truth values.

## Materials and methods

### Acquisition of 2D cine MR images using the 1.5 T MR-linac

In contrast to conventional radiotherapy, the MRgRT workflow involves acquiring pre-treatment MR images, which are registered with a reference image set. This process allows for the modification of the reference treatment plan to accommodate daily variations in anatomical position. Additionally, MRgRT using the 1.5T MR-linac (Elekta Unity) enables the acquisition of 2D cine MR images, which are obtained using a 2D balanced fast field echo (bFFE) sequence provided by Elekta for use during treatment. These cine MR images have a temporal resolution of 0.2 s, and each imaging plane is acquired in an interleaved fashion. The number and types of imaging planes (coronal, sagittal, axial, or a combination thereof) can be selected by the user. For this study, we utilized the 2D cine MR images obtained during MRgRT sessions on the 1.5T MR-linac system at our hospital. Several randomly selected cases from various treatment sites, including prostate, liver, bladder, and others, were used to develop an in-house software package. Additionally, five cases of prostate cancer were utilized to verify the developed software package. A total of 28 imaging sessions were included, with each session yielding approximately 300 to 3,500 cine MR images. This study was approved by the institutional review board (IRB) of Gangnam Severance Hospital (IRB approval no. 3-2022-0413), and the requirement for informed consent was waived owing to the retrospective study design. Furthermore, all methods were performed in accordance with the relevant guidelines and regulations.

### File conversion: binary file format into metadata or DICOM file format

We developed in-house software using MATLAB (MathWorks, Natick, MA, USA) to convert the binary file format of 2D cine MR images acquired through treatment session manager (TSM)^21^ of MOSAIQ version 2.83 (Elekta AB, Stockholm, Sweden) during Unity MR-linac treatment into metadata files, which are widely used in open-source software packages for image registration, such as ITK^22^ plastimatch^23^ and elastix^24^. Additionally, the software converts the binary file format into DICOM format, which is the standard format for medical imaging data such as X-rays, CT scans, MRI scans, and ultrasound images^25^.

All cine data required for the conversion are stored in the Data Server Appliance (DSA). The DSA can be accessed via the Windows Desktop Viewer using a known DSA IP address. Within the DSA, all cine data are organized into patient-specific directories, each containing subfolders with cine images for each treatment fraction. These are sorted based on unique identifiers (UIDs) that are assigned by the TSM and used by the MONACO TPS (Elekta AB, Stockholm, Sweden).

**Fig. 1:**
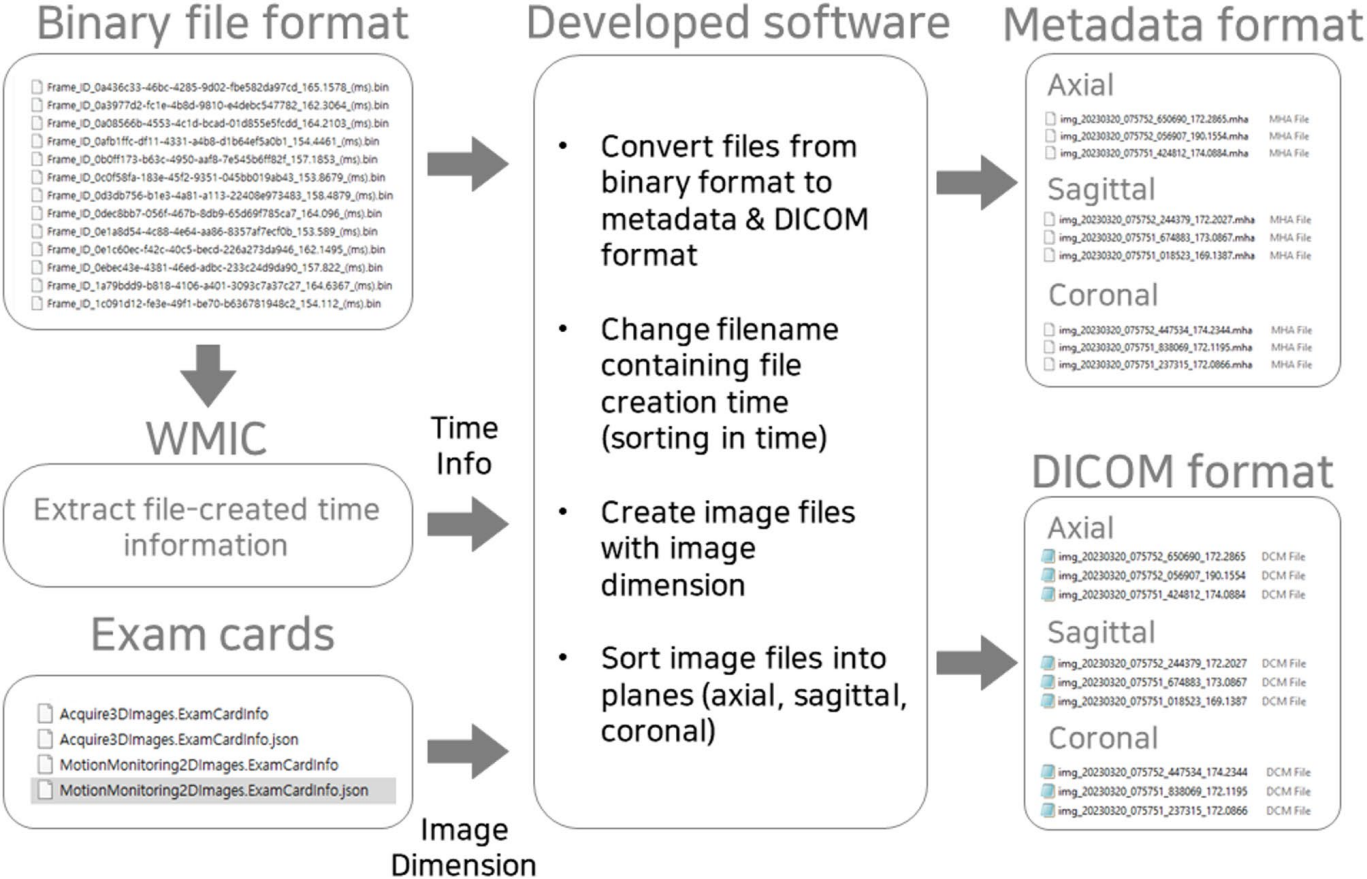
Overall workflow of converting cine MR images from binary file format into metadata and DICOM file formats.

The overall workflow of the file conversion process is shown in Fig. 1. The following information is necessary for this process: header length, data type (bit length), image dimensions (number of pixels in both directions), and pixel spacing. First, among these cine MR image parameters, the header length and data type were empirically determined by trial and error. We initially focused on the most commonly used data types such as 8-bit, 16-bit, and 32-bit. And for header length, we specifically examined sections where the representation format changed. Therefore, the header length was found to be 4,084, assuming an 8-bit data type. In-house MATLAB software was developed to read the image data as unsigned 16-bit integers.

Second, the image dimensions and pixel spacing were obtained from a JSON file (MotionMonitoring2DImages.ExamCardInfo.json) located in the ExamCards folder of each motion monitoring session. Specifically, the number of pixels in the 2D cine MR image is recorded with the names “Rows” and “Columns,” and the image sizes are recorded with the names “SliceDimensionXInmm” and “SliceDimensionYInmm” in the JSON file. The pixel spacing was calculated using these parameters (number of pixels and image size). Moreover, the number of pixels obtained from the cine imaging acquisition parameter file determined the data length when the image data were read in an unsigned 16-bit integer format using the developed software.

Third, the 2D cine MR images were acquired at the center of the target structure, so the centroid coordinates of the converted 2D cine MR images were assigned to the centroid coordinates of the target structure. This information was obtained from a JSON file in the BinaryMasks folder of the motion monitoring session. We confirmed that this centroid information exactly matched the target centroid structure displayed in the MONACO TPS version 5.51.11.

Furthermore, the cine MR image files were renamed so that the modified file names contained information on file creation time, thereby ordering the images in time. The original binary files did not explicitly provide any time-related information. The Windows Management Instrumentation command-line utility was used to extract image creation time information. Because the cine MR images were acquired with a high frame rate (~ 5 frames/s), resulting in multiple cine MR acquisitions in a second, the file creation time information had to be obtained on the millisecond time scale.

### Identifying the image planes of the converted images

**Fig. 2:**
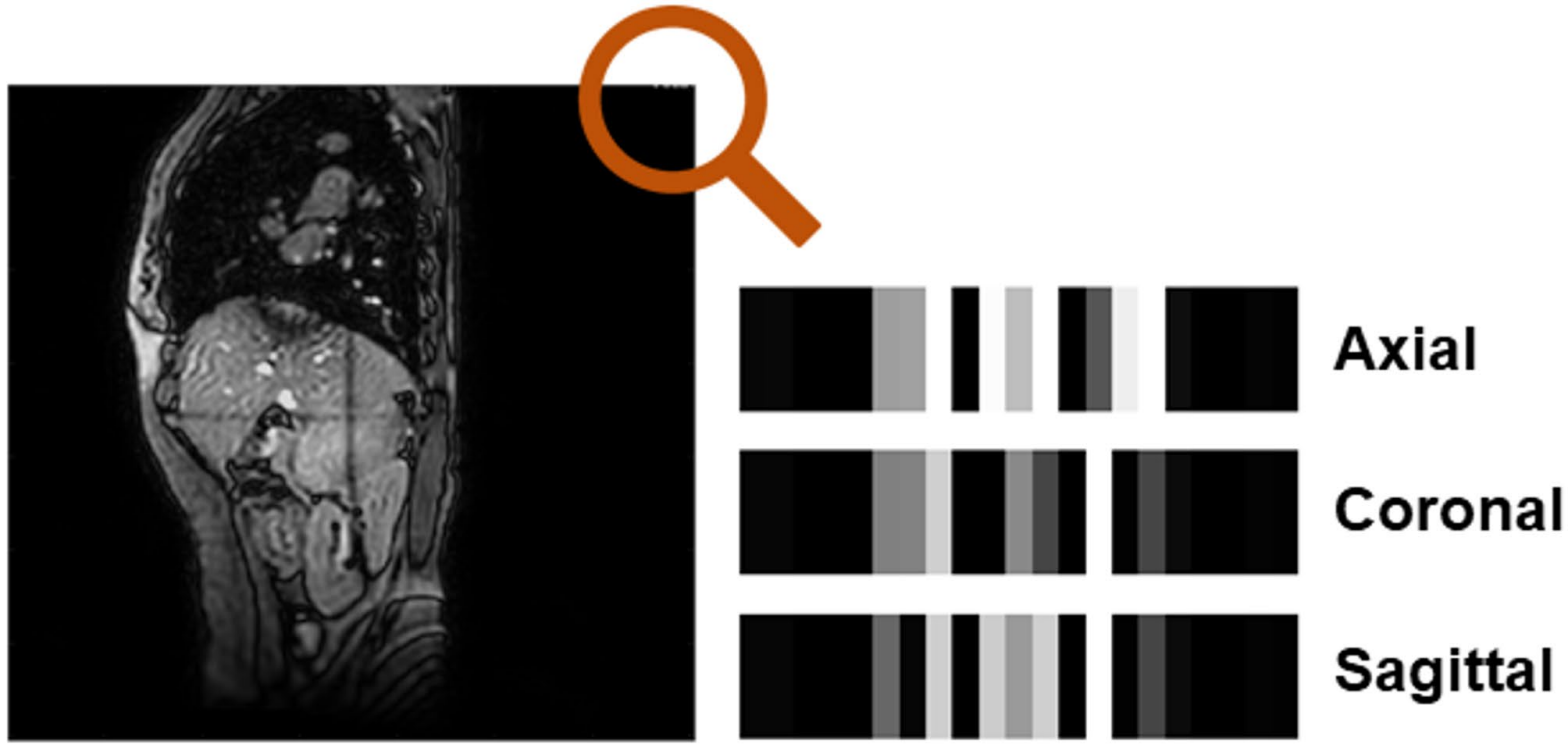
Illustration of plane-specific intensity patterns in the upper right corner of the cine MR image, used to automatically identify the imaging plane of each converted image.

An automatic algorithm was developed to classify planes according to their orientations (coronal, sagittal, and axial) using the information stored in the top-right corner of each cine MR image (Fig. 2). While the intensity values in this information were varied for each monitoring session, a consistent pattern for each plane was found. The cine MR images extracted from the binary files were shifted by a certain number of pixels (22 pixels) so that the final images could be correctly centered.

Additionally, in order to register 2D cine MR images with 3D MR images or target contour structures, it is necessary to align the coordinates of each image. Since 2D cine MR images were acquired from the center of the target contour structure, the center coordinates of the 2D cine MR images should be aligned with the center coordinates of the target structure. To achieve this, the center coordinates of the target structure were obtained from the JSON file in the BinaryMasks folder and set as the center of the 2D cine MR images.

The plane-identification accuracy of the developed algorithm was verified over several sessions using five prostate cancer patients who received definitive radiotherapy using Unity-based MRgRT. For these five patients, a total of 25 imaging sessions were stored; the cine MR images were acquired while considering patient comfort. Approximately, 300 to 3,500 cine MR images were obtained for each session. Therefore, a total of 36,762 cine MR images were subjected to file-format conversion and imaging plane classification. After conversion and classification, the accuracy of the plane assignment was evaluated by visually inspecting the images for each plane and manually identifying any incorrectly classified cine MR images.

### Geometric verification of converted cine MR images: without motion

The geometric accuracy of the converted cine MR images was verified by acquiring cine MR images from an MR-compatible phantom: the ZEUS MRgRT motion management QA phantom (CIRS Inc., Norfolk, VA, USA). The 2D cine MR images were acquired at the center of the moving target in the phantom and converted into metadata format using in-house conversion software, as described above. The converted 2D images were manually registered with 3D T1 MR images using 3D Slicer software^22^an open-source software application for the visualization and analysis of medical image computing datasets, in order to visually compare their geometry.

**Fig. 3:**
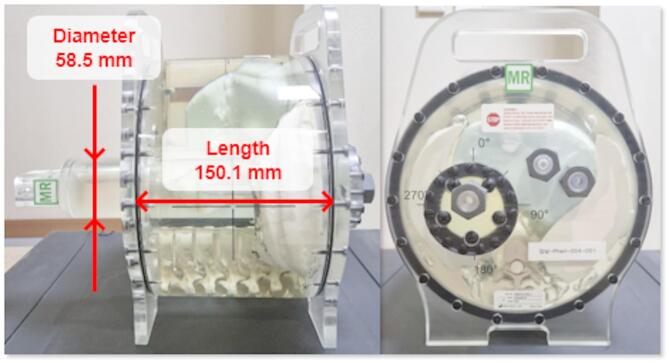
ZEUS MRgRT motion management QA phantom with the dimensions-of-interest presented.

Several dimensions were measured on the cine MR images of the phantom using 3D Slicer and compared with the known dimensions. Specifically, the inner diameter of the cylindrical rod in the phantom (58.5 mm) and the inner length of the phantom body (150.1 mm) were each measured twice respectively from the cine MR images of the phantom (Fig. 3).

### Geometric verification of cine MR images: with simulated motions

The geometric accuracy of the converted cine MR images was further investigated by acquiring cine MR images of a phantom with simulated clinically possible motions. This experiment was designed to evaluate the motion-capturing accuracy of cine MR imaging for two clinical scenarios: (1) respiratory-related motion of a moving target in the thorax of the patient and (2) translations of a region including vertebral bodies.

**Fig. 4:**
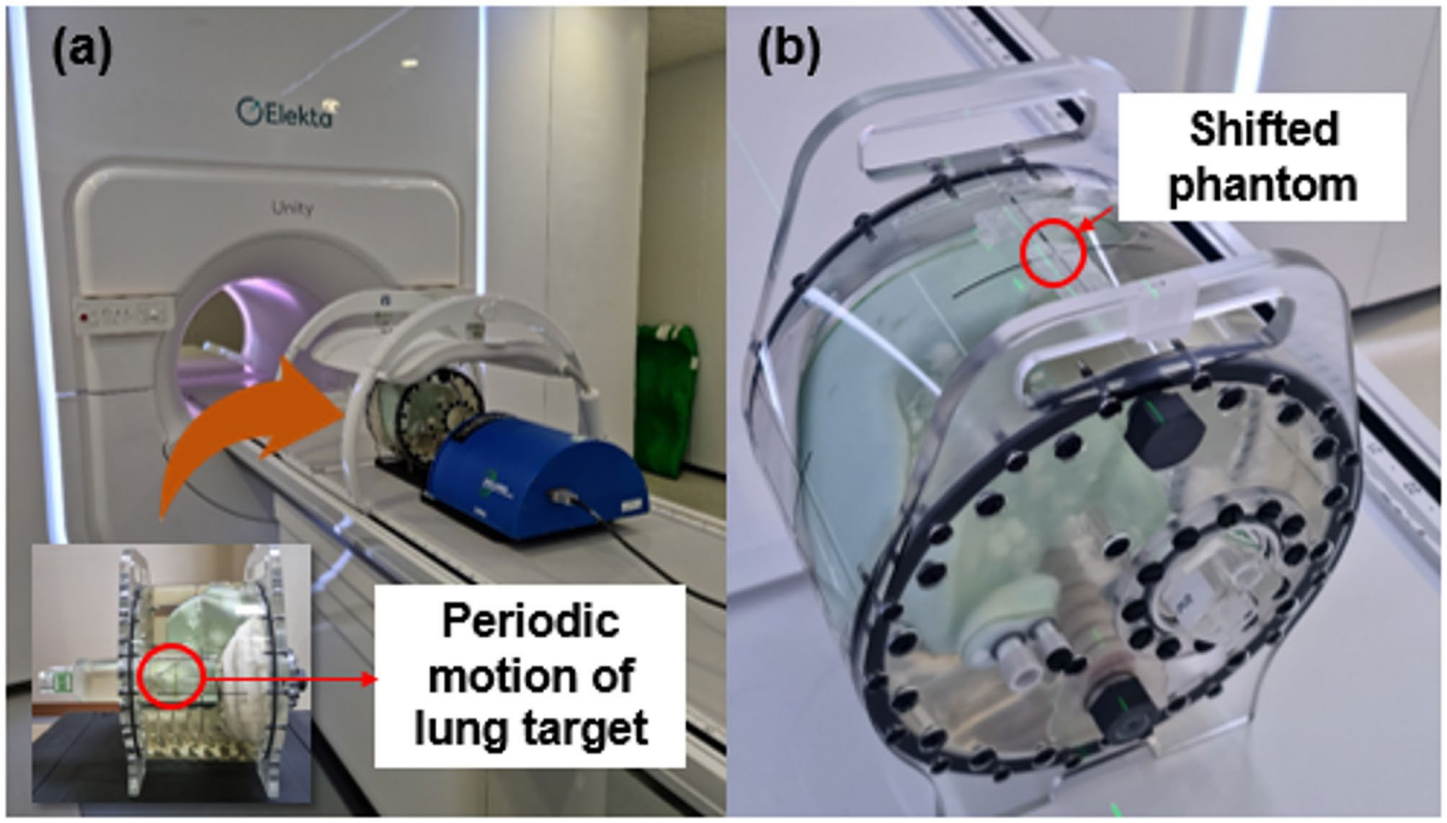
Experimental setup for two simulated motions: (**a**) respiratory motion of a moving target in the lung, (**b**) translational motion of the thorax region, including vertebral bodies, by shifting the phantom.

As shown in Fig. 4a, the motion of a moving target owing to respiration in the lung was simulated by designing the periodic motion of a cylindrical rod in the motion management QA phantom. The cylindrical rod was designed to move at amplitudes of 5, 10, 15, and 20 mm every 4 s. The acquired cine MR images were converted into the DICOM file format.

The converted images were imported into MIM software version 7.2.10 (MIM Software Inc., Cleveland, OH, USA), where the moving target was manually contoured in the 2D coronal images, and the centroid coordinates were automatically calculated. The centroid coordinates of the target were first obtained from converted MM images when the phantom was stationary. Using these reference coordinates, we then extracted the centroid coordinates from images acquired during reciprocating phantom motion. The mean amplitude and standard deviation of the target motion were calculated and compared with the ground truth motion amplitude.

The translational motion of the vertebral body was simulated by manually shifting the motion management QA phantom on the couch. The reference position was set up using a sagittal laser, and both 3D (T1 sequence) and 2D cine-MR images were acquired at that position. The phantom was then manually shifted by 0.5, 1, 2, 3, and 5 mm using a sagittal laser, as shown in Fig. 4b, and 3D and 2D cine MR images were acquired. The 2D cine MR images were converted to the DICOM format using in-house software. The 3D and converted 2D cine MR images of the shifted QA phantom were registered based on the MR images of the non-shifted phantom using the MIM software. These 3D-3D and 2D-2D registrations were performed by aligning the vertebral bodies in the phantom. The offsets between the non-shifted and shifted 2D cine images were compared with those of the 3D MR images.

## Results

### File conversion and plane sorting

**Fig. 5:**
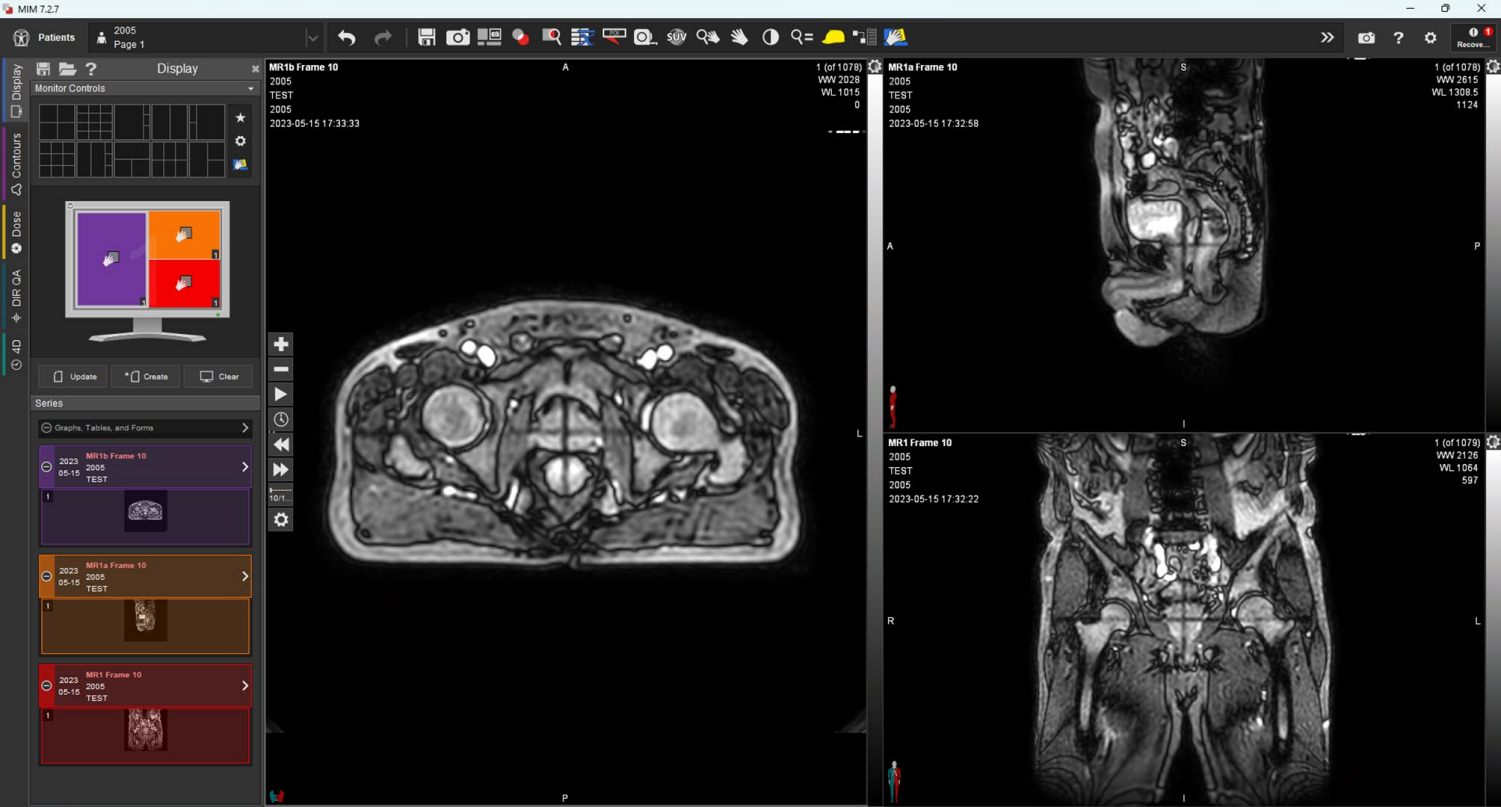
Converted 2D cine MR images acquired during MR-guided radiation treatment of a prostate cancer patient displayed in MIM software.

**Fig. 6:**
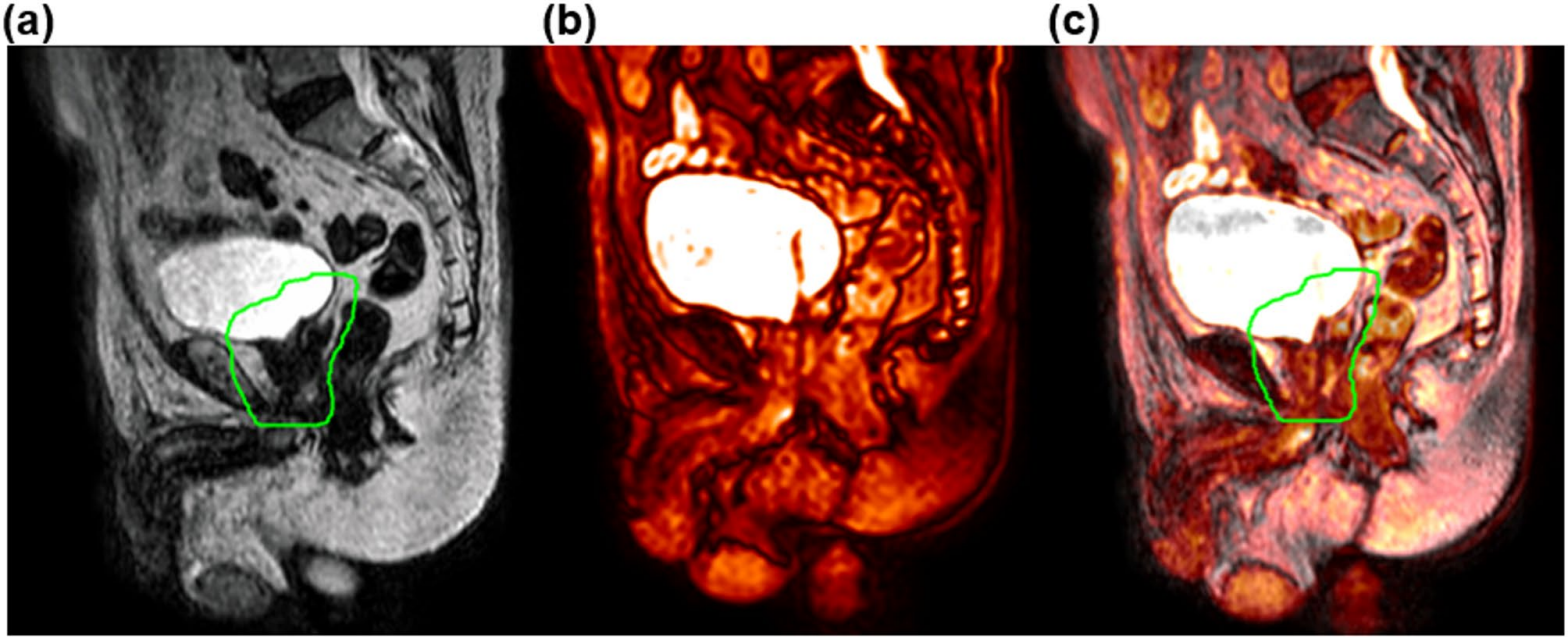
(**a**) Daily MR images included PTV structure (green line), (**b**) the converted 2D cine MR images, and (**c**) registration of two images in the sagittal planes.

The cine MR images, which were originally saved in an unknown binary file format, were successfully converted into the DICOM file format, making it possible to import them into MIM software (see Fig. 5). The MATLAB scripts for 2D cine MR images conversion were deposited in a public GitHub repository (https://github.com/jwsung813/MM-to-DCM-conversion) and protocols.io (https://dx.doi.org/10.17504/protocols.io.ewov11oq7vr2/v1). In addition, all 36,762 cine MR images from the five patients with prostate cancer were correctly sorted into three imaging planes (axial, sagittal, and coronal). Figure 6 shows the registered image (Fig. 6c) of the daily MR image (Fig. 6a) acquired before treatment and the converted 2D cine MR image (Fig. 6b) acquired on the same day, with their center coordinates aligned. The images demonstrate an accurate match. This allows not only for the verification that the target is accurately within the PTV during beam delivery but also enables intra-fractional motion analysis.

### Geometric verification of cine MR image: without motion

**Fig. 7:**
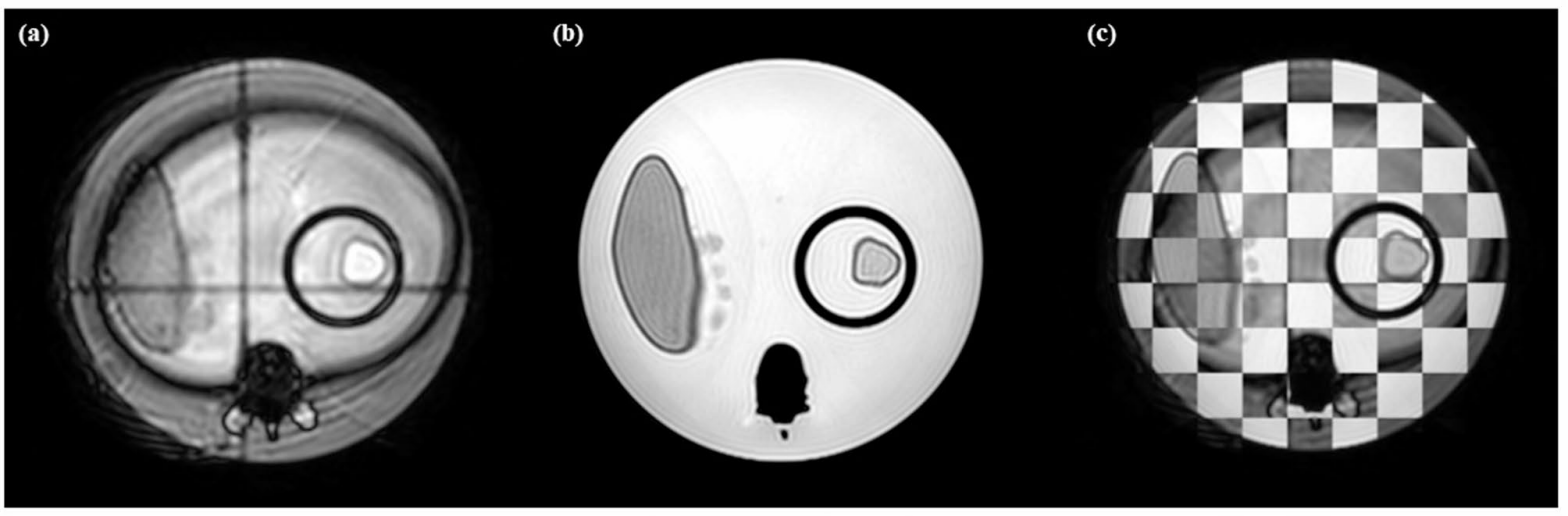
Manual image registration results of a (**a**) converted 2D cine MR image and (**b**) 3D T1 image, and (**c**) comparison between the two images as a checkerboard image using 3D Slicer.

The inner diameter of the cylindrical rod and inner length of the phantom body in the motion management phantom were measured and compared to the known ground truth dimensions, as summarized in Table 1. The deviations of the measured dimensions from the ground truth values were 0.2 mm and − 0.5 mm for the inner diameter of the cylindrical rod and the inner length of the phantom body, respectively. Furthermore, an axial image of the 3D MR scan of the phantom was compared with an axial cine MR image of the phantom, as shown in Fig. 7.

**Table 1:** Results of geometric verification of converted 2D cine MR images using a motion management MR-compatible Phantom without motion.

Item	Measured (mm)			Ground truth (mm)	Error (mm)
	1	2	Mean		
Inner diameter of cylindrical rod	58.5	58.9	58.7	58.5	0.2
Length of phantom body	149.3	149.8	149.6	150.1	–0.5

### Geometric verification of cine MR image: with simulated motions

To verify the converted 2D cine MR images, the respiratory amplitudes measured using the converted 2D cine MR images were compared with known ground truth. For each of the respiratory motions, the difference between the amplitude measured using cine MR images and the ground truth was − 0.34 mm on average, and the errors for each amplitude are shown in Table 2.

**Table 2:** Differences between the respiratory amplitudes from cine MR images and the ground truth motions.

Ground truth amplitude (mm)	Amplitude from 2D cine MR (mean/standard deviation, mm)	Error (mm)
5	4.7 ± 0.2	–0.3
10	9.7 ± 0.5	–0.3
15	14.9 ± 0.4	–0.1
20	19.4 ± 0.6	–0.6

Furthermore, the shifted distances of the phantom spine captured by the 2D cine MR images were compared with those captured by the 3D MR images. The differences between the shifted distances measured using the converted 2D cine MR and 3D MR images are summarized in Table 3. All errors were less than 0.2 mm.

**Table 3:** Differences between the translation motions of the Phantom spine measured by using 2D cine MR and the 3D MR images.

Expected phantom shift (mm)	3D MR (mm)	2D cine MR (mm)	Error (mm)
0.5	0.00	0.00	0
1.0	0.05	0.00	–0.05
2.0	1.54	1.49	–0.05
3.0	2.60	2.75	0.15
5.0	4.78	4.60	–0.18

## Discussion

Unity MR-linac is capable of acquiring high-quality 1.5T MR images and offers the advantage of utilizing 2D cine MR images in all three directions: coronal, sagittal, and axial. The availability of cine MR images in all directions allows for a more thorough evaluation of target motion, facilitating the optimization of target margins. We believe that this investigation will make a meaningful contribution to the radiation therapy community as it provides a software tool for processing cine MR images in an unknown file format. By using the publicly available software tools developed in this study, other institutions that are currently using and will use the 1.5 T MR-linac can access the cine MR images of MRgRT patients more easily. Processing cine MR images and incorporating the analysis results of during-treatment patient motions into clinical practice will lead to a better understanding of the characteristics of in-treatment patient motions and therefore improve the quality of patient treatment. Furthermore, easy access to cine MR images can promote research efforts to develop cine MR-based image processing techniques, including but not limited to real-time motion-tracking algorithms.

In addition to the release of the developed software, we evaluated the geometric accuracy of the converted cine MR images and the ability of cine MR imaging to capture simulated motions that are likely to occur during radiation treatment of lung tumors and vertebral bodies. First, by acquiring cine MR images of an MR-compatible phantom without motion and comparing several dimensions between 2D cine MR images and 3D MR images, the geometric accuracy was evaluated. Submillimeter differences between the ground truth and measured dimensions demonstrate that the developed algorithm can convert binary format files into a readable image format with appropriate geometric scaling. Second, the motions introduced into the lung target and the vertebral body in the phantom were successfully captured. Although manual contouring was performed to analyze the motions in the phantom, the clarity of the target’s boundaries on the 2D cine MR images suggests that there would be minimal uncertainty regarding the position and size of the contoured target. Applying an automatic image registration algorithm on the cine MR images will be an interesting future research topic to examine the feasibility of automatic motion tracking using cine MR imaging.

However, 2D cine MR images inherently have issues due to the subsequent acquisition of 2D MR images in multiple planes. Dark-band artifacts were clearly observed when 2D cine MR images were acquired in all three planes. These artifacts appear as dark signal lines on 2D cine MR images owing to the intersections of nonparallel imaging planes in rapidly acquired MR images^26,27^. Although this study aligned the images by matching their center coordinates or manually, dark-band artifacts can interfere with automatic registration in general image registration algorithms. The dark signal line caused by a dark-band artifact has a significantly different pixel intensity than those in other regions, resulting in high image intensity gradients along the artifact, which can bias the behavior of intensity-based image registration algorithms. One approach for reducing the effect of dark-band artifacts is to acquire 2D images with one or two planes. Another approach is to analyze the signal loss profile in the affected area. In a previously published study, MR images of a water tank were acquired using the same 2D cine MR sequence to characterize the signal loss profile. Then, the signal in the affected regions of the patient images was compensated by multiplying it with the inverse signal loss profile^28^.

This study primarily focused on the development and initial validation of the conversion software. As mentioned above, the 2D cine MR data acquired during treatment using MOSAIQ is stored in an unreadable binary format, which cannot be accessed by commercial software or even within MOSAIQ itself after treatment. As a result, direct comparison between the converted data and the original data for validation purposes is not feasible. Despite this limitation, the successful visualization of the converted images in both the open-source software 3D Slicer and the commercial software MIM demonstrates the practical feasibility of the software for clinical and research use.

In future studies, we plan to expand the developed software into a tool capable of analyzing organ motion during radiation delivery using 2D cine MR images. To achieve this, we will implement an image preprocessing function that automatically corrects dark-band artifacts and incorporate various features, such as image registration with different imaging modalities (e.g., CT, MRI), extraction of three-axis motion graphs, and volumetric motion analysis using deformable image registration. Once development is complete, the software will be made available as open source, with the goal of providing practical support for both clinical and research applications among users of the Elekta Unity system. The proposed cine MR conversion software can also be used to develop more advance motion analysis methods. For instance, the current version of clinical real-time tracking software is limited to analyzing motion only within the tumor region and does not provide motion information for surrounding tissues. A deep learning-based motion analysis approach has the potential to evaluate during-treatment motion in both tumor and multiple normal tissue regions, while minimizing latency.

## Conclusion

We developed a software which converts 2D cine MR images stored in a binary format into a commonly used file format and verified the accuracy of the converted images. This software has been shared with the public through a GitHub repository and protocols.io. It is expected to assist 1.5 T MR-linac users in understanding internal organ motion during treatment.

## Supplementary Information

Below is the link to the electronic supplementary material.

Supplementary Material 1

## Data Availability

The data and materials in the current study are available from the corresponding author on reasonable request. The MATLAB scripts for MM conversion have been deposited in a public GitHub repository (https://github.com/jwsung813/MM-to-DCM-conversion) and protocols.io (https://dx.doi.org/10.17504/protocols.io.ewov11oq7vr2/v1).
