# Supplementary material for: Development of in-house software to process real-time cine magnetic resonance images acquired during 1.5 T MR-guided radiation therapy
Source: Sci Rep. 2025 Aug 12;15:29515. doi: 10.1038/s41598-025-15107-4 (PMC12343943; doi:10.1038/s41598-025-15107-4)
Supplement: Supplementary file 1 — Supplementary Material 1 [file 41598_2025_15107_MOESM1_ESM.pdf]

Main MatLAB script

```
clear all
close all
clc

%% change the  binary files name

WorkingFolder = input('Write the path of patient folder:\n\nex)
E:\MM\1.3.\ ....\n\n:','s')
folder_TwoDImages = sprintf('%s\\TwoDImages',WorkingFolder);
binaryfiles = dir(sprintf('%s\\Frame_ID_*.bin',folder_TwoDImages));

for ff = 1:size(binaryfiles)
    filename = binaryfiles(ff).name;

    strsplit_filename = strsplit(filename,'_');
    time_info = strsplit_filename{4};

    filepath = sprintf('%s\\%s',binaryfiles(ff).folder,filename);
    filepath_rep = strrep(filepath,'\','\\');

    command_wMIC = sprintf('wmic datafile where name="%s" get lastmodified |
findstr /brc:[0-9]',filepath_rep);
    [~, modifiedTime] = system(command_wMIC);

    str_date = modifiedTime(1:8);
    str_time = modifiedTime(9:14);
    str_microseconds = modifiedTime(16:21);

    filename_renamed =
sprintf('file_%s_%s_%s_%s.bin',str_date,str_time,str_microseconds,time_info);
    filepath_renamed = sprintf('%s',filename_renamed);

    command = sprintf('rename "%s" "%s"',filepath,filepath_renamed);
    system(command);
end


%% Detection of Discontinuous Time Intervals

binaryfiles = dir(sprintf('%s\\file_*.bin',folder_TwoDImages));
acquisition_timepoints = zeros(size(binaryfiles,1),1);

for ff = 1:size(binaryfiles)
    filename = binaryfiles(ff).name;

    strsplit_filename = strsplit(filename,'_');

    str_hhmmss = strsplit_filename{3};
    str_ms = strsplit_filename{4};

    hh = str2double(str_hhmmss(1:2));
    mm = str2double(str_hhmmss(3:4));
    ss = str2double(str_hhmmss(5:6));
    ms = str2double(str_ms);      % in microsecond
```

```
    % calculate timepoint in milliseconds
    timestamp_in_millisecond = 60*60*1000*hh + 60*1000*mm + 1000*ss + ms/1000;

    acquisition_timepoints(ff,1) = timestamp_in_millisecond;
end

acquisition_timeinterval = abs(diff(acquisition_timepoints));

[max_interval, index] = max(acquisition_timeinterval);
[maximum5intervals, index1] = maxk(acquisition_timeinterval,5);

aa= maximum5intervals > 10^5;
aaIndex = find(aa==1);
aaRealIndex=index1(aaIndex);

if max_interval > 10^5
    filename_discontinuity = sprintf('%s\\discontinuity.txt',folder_TwoDImages);
    fid = fopen(filename_discontinuity,'w');
    for bb = 1:length(aaRealIndex)
        filename_temp = binaryfiles(aaRealIndex(bb)).name;
        strsplit_filename = strsplit(filename_temp,'_');
        str_yyyymmdd = strsplit_filename{2};
        str_hhmmss = strsplit_filename{3};
        str_ms = strsplit_filename{4};
        fprintf(fid,'%s_%s_%s\n',str_yyyymmdd,str_hhmmss,str_ms);
    end
    fclose(fid);
end


if exist ('filename_discontinuity','var')
    warning('it is interval in here, Please separate the MM files btw intervals')
    pause
end

%% Classification of axial coronal sagittal

binaryfiles = dir(sprintf('%s\\file_*.bin',folder_TwoDImages));
[~,~,~,ImageSizeRow,ImageSizeColumn]= GetMotionMonitoringInfo(WorkingFolder);
phantom_ID = zeros(ImageSizeRow, size(binaryfiles,1));

for ff=1:size(binaryfiles)
    filename_temp = binaryfiles(ff).name;
    fprintf('Fraction: %s\n',filename_temp)
    foldername_temp = binaryfiles(ff).folder;
    filepath_temp = sprintf('%s\\%s',foldername_temp, filename_temp);
    mm_image = GetMotionMonitoringImage(filepath_temp, WorkingFolder);


    image(mm_image)
    phantom_ID(:,ff)=mm_image(1:ImageSizeRow,1);
end

ImageSizeRow_axial=phantom_ID(ImageSizeRow-5,:);
ImageSizeRow_sagittal=phantom_ID(ImageSizeRow-13,:);
ImageSizeRow_coronal=phantom_ID(ImageSizeRow-9,:);
ImageSizeRow_axial_factors=unique(ImageSizeRow_axial);
ImageSizeRow_sagittal_factors=unique(ImageSizeRow_sagittal);
```

```
ImageSizeRow_coronal_factors=unique(ImageSizeRow_coronal);

if length(ImageSizeRow_sagittal_factors) > 2
    warning('separate the binary files')
    return;
elseif length(ImageSizeRow_coronal_factors) > 2
    warning('separate the binary files')
    return;
elseif length(ImageSizeRow_axial_factors) > 2
    warning('separate the binary files')
    return;
end


if sum(ImageSizeRow_axial_factors(1,1)==ImageSizeRow_axial) >
sum(ImageSizeRow_axial_factors(1,2)==ImageSizeRow_axial)
    axialFactor=ImageSizeRow_axial_factors(1,2)
else
    axialFactor=ImageSizeRow_axial_factors(1,1)
end

if sum(ImageSizeRow_coronal_factors(1,1)==ImageSizeRow_coronal) >
sum(ImageSizeRow_coronal_factors(1,2)==ImageSizeRow_coronal)
    coronalFactor=ImageSizeRow_coronal_factors(1,2)
else
    coronalFactor=ImageSizeRow_coronal_factors(1,1)
end

if sum(ImageSizeRow_sagittal_factors(1,1)==ImageSizeRow_sagittal) >
sum(ImageSizeRow_sagittal_factors(1,2)==ImageSizeRow_sagittal)
    sagittalFactor=ImageSizeRow_sagittal_factors(1,2)
else
    sagittalFactor=ImageSizeRow_sagittal_factors(1,1)
end


folder_TwoDImagesRenamed_axial = sprintf('%s\\axial',folder_TwoDImages);
folder_TwoDImagesRenamed_sagittal = sprintf('%s\\sagittal',folder_TwoDImages);
folder_TwoDImagesRenamed_coronal = sprintf('%s\\coronal',folder_TwoDImages);


if ~exist(folder_TwoDImagesRenamed_axial,'dir')
    mkdir(folder_TwoDImagesRenamed_axial)
end

if ~exist(folder_TwoDImagesRenamed_sagittal,'dir')
    mkdir(folder_TwoDImagesRenamed_sagittal)
end

if ~exist(folder_TwoDImagesRenamed_coronal,'dir')
    mkdir(folder_TwoDImagesRenamed_coronal)
end

for ff=1:size(binaryfiles)
    filename_temp = binaryfiles(ff).name;
    fprintf('Fraction: %s\n',filename_temp)
    foldername_temp = binaryfiles(ff).folder;
    filepath_temp = sprintf('%s\\%s',foldername_temp, filename_temp);
```

```
    mm_image = GetMotionMonitoringImage(filepath_temp, WorkingFolder);
    if mm_image(ImageSizeRow-9,1) == coronalFactor
        folder_to_sort = sprintf('%s\\coronal',folder_TwoDImages);
        filepath_new = sprintf('%s\\%s',folder_to_sort,filename_temp);
        movefile(filepath_temp,filepath_new)
    elseif mm_image(ImageSizeRow-13,1) == sagittalFactor
        folder_to_sort = sprintf('%s\\sagittal',folder_TwoDImages);
        filepath_new = sprintf('%s\\%s',folder_to_sort,filename_temp);
        movefile(filepath_temp,filepath_new)
    elseif mm_image(ImageSizeRow-5,1) == axialFactor
        folder_to_sort = sprintf('%s\\axial',folder_TwoDImages);
        filepath_new = sprintf('%s\\%s',folder_to_sort,filename_temp);
        movefile(filepath_temp,filepath_new)
    end

end

%%  Convert binary files to MHA files

ListPlanes = {'coronal','sagittal','axial'};
offset = [0.0, 0.0, 0.0];

% image information
[SliceDimensionXInmm, SliceDimensionYInmm, SliceDimensionZInmm, Rows, Columns] = 
GetMotionMonitoringInfo(WorkingFolder);

spacing = zeros(1,3);
spacing(1) = SliceDimensionXInmm/Rows;
spacing(2) = SliceDimensionYInmm/Columns;
spacing(3) = SliceDimensionZInmm;

% folder containing two-dimensional motion monitoring images
folder_TwoDImages_mha = sprintf('%s\\TwoDImages_mha',folder_TwoDImages);
folder_TwoDImages_mha_axi = 
sprintf('%s\\TwoDImages_mha\\axial',folder_TwoDImages);
folder_TwoDImages_mha_sag = 
sprintf('%s\\TwoDImages_mha\\sagittal',folder_TwoDImages);
folder_TwoDImages_mha_cor = 
sprintf('%s\\TwoDImages_mha\\coronal',folder_TwoDImages);

if ~exist(folder_TwoDImages_mha,'dir')
    mkdir(folder_TwoDImages_mha)
end
if ~exist(folder_TwoDImages_mha_axi,'dir')
    mkdir(folder_TwoDImages_mha_axi)
end
if ~exist(folder_TwoDImages_mha_sag,'dir')
    mkdir(folder_TwoDImages_mha_sag)
end
if ~exist(folder_TwoDImages_mha_cor,'dir')
    mkdir(folder_TwoDImages_mha_cor)
end

for plane = 1:3
    folder_TwoDImages_plane = 
sprintf('%s\\%s',folder_TwoDImages,ListPlanes{plane});
    binaryfiles = dir(sprintf('%s\\file_*.bin',folder_TwoDImages_plane));
```

```
    for ff = 1:size(binaryfiles,1)
        filename_bn = binaryfiles(ff).name;
        filepath_bn = sprintf('%s\\%s',binaryfiles(ff).folder,filename_bn);

        % folder for mha file
        folder_TwoDImages_mha_plane =
sprintf('%s\\%s',folder_TwoDImages_mha,ListPlanes{plane});
        filepath_mha =
sprintf('%s\\img_%s.mha',folder_TwoDImages_mha_plane,filename_bn(6:end-4));

        mm_image_2D = GetMotionMonitoringImage(filepath_bn, WorkingFolder);

        mm_image_3D = zeros([size(mm_image_2D), 1]);
        mm_image_3D(:,:,1) = mm_image_2D;

         writemha2D(filepath_mha,mm_image_3D,offset,spacing,'ushort');
    end
end

%% Convert MHA files to DCM

% MM center
[MMcenterX, MMcenterY, MMcenterZ]  = GetMMcenterInfo(WorkingFolder);

ListPlanes = {'coronal','sagittal','axial'};
PatientID = input('Patient ID: ','s')
PatientName = input('Patient name: ','s')

for plane = 1:3
    input_folder = sprintf('%s\\%s',folder_TwoDImages_mha, ListPlanes{plane});
    output_folder = sprintf('%s\\TwoDImages_dcm\\%s',folder_TwoDImages,
ListPlanes{plane});

    if ~exist(output_folder,'dir')
        mkdir(output_folder)
    end

    files = dir(sprintf('%s\\*.mha',input_folder));

    for ff = 1:size(files,1)
        filename_mha = sprintf('%s\\%s',files(ff).folder,files(ff).name);

        % read mha files
        image_header = mha_read_header(filename_mha);
        image_origin = image_header.Offset;
        image_spacing = image_header.PixelDimensions;
        image_size = image_header.Dimensions;
        image = mha_read_volume(image_header);
        image = permute(image,[2 1 3]);

        % write dicom files
        filename_temp = files(ff).name;
        filename_dcm = sprintf('%s\\%s.dcm',output_folder,filename_temp(1:end-4));

        dicomwrite(image,filename_dcm)

        % write dicom information
        info = dicominfo(filename_dcm);
```

```
        if ff == 1
            SeriesInstanceUID = info.SeriesInstanceUID;
        else
            info.SeriesInstanceUID = SeriesInstanceUID;
        end

        info.PatientID =  PatientID;
        info.PatientName = PatientName;
        info.PixelSpacing = image_spacing(1:2);
        info.SliceThickness = image_spacing(3);
        info.Modality = 'MR';
        if plane == 1
            info.SeriesDescription = 'CORONAL';
            info.ImageType = 'PRIMARY\CORONAL';
            info.ImageOrientationPatient = [1, 0, 0, 0, 0, -1];
            ImagePositionPatient = zeros(1,3);
            ImagePositionPatient(1) =  -image_spacing(1)*(image_size(1) -
1)/2+MMcenterX;
            ImagePositionPatient(2) =  -MMcenterZ;
            ImagePositionPatient(3) =  image_spacing(1)*(image_size(1) - 1)/2-
MMcenterY;
            info.ImagePositionPatient = ImagePositionPatient;
        elseif plane == 2
            info.SeriesDescription = 'SAGITTAL';
            info.ImageType = 'PRIMARY\SAGITTAL';
            info.ImageOrientationPatient = [0, 1, 0, 0, 0, -1];
            ImagePositionPatient = zeros(1,3);
            ImagePositionPatient(1) =  MMcenterY;
            ImagePositionPatient(2) =   -image_spacing(1)*(image_size(1) - 1)/2-
MMcenterZ;
            ImagePositionPatient(3) =   image_spacing(1)*(image_size(1) -
1)/2+MMcenterX;
            info.ImagePositionPatient = ImagePositionPatient;
        elseif plane == 3
            info.SeriesDescription = 'AXIAL';
            info.ImageType = 'PRIMARY\AXIAL';
            info.ImageOrientationPatient = [1, 0, 0, 0, 1, 0];
            ImagePositionPatient = zeros(1,3);
            ImagePositionPatient(1) = -image_spacing(1)*(image_size(1) -
1)/2+MMcenterX;
            ImagePositionPatient(2) = -image_spacing(1)*(image_size(1) -
1)/2+MMcenterY;
            ImagePositionPatient(3) =  -MMcenterZ;
            info.ImagePositionPatient = ImagePositionPatient;

        end

        dicomwrite(image,filename_dcm,info,"CreateMode","copy");

        info_read = dicominfo(filename_dcm);

    end
end

%% Coordinate for each plane

MMcenter = [MMcenterX, MMcenterY, MMcenterZ]
MMaxial = [MMcenterX, MMcenterY, -MMcenterZ]
```

```
MMsagittal = [MMcenterY, -MMcenterZ, MMcenterX]
MMcoronal = [MMcenterX, -MMcenterZ, -MMcenterY]
```

Function script 1

```
function [SliceDimensionXInmm, SliceDimensionYInmm, SliceDimensionZInmm, ...
    Rows, Columns] = GetMotionMonitoringInfo(FractionFolder)

folder_ExamCards = sprintf('%s\\ExamCards',FractionFolder);

strings_info = {'"SliceDimensionXInmm":'; ...
    '"SliceDimensionYInmm":'; ...
    '"SliceDimensionZInmm":'; ...
    '"SliceThicknessInmm":'; ...
    '"Rows":'; ...
    '"Columns":'};

if exist(folder_ExamCards,'dir')
    filename_MM_ExamCardInfo =
sprintf('%s\\MotionMonitoring2DImages.ExamCardInfo.json',folder_ExamCards);

    if exist(filename_MM_ExamCardInfo,'file')

        fid = fopen(filename_MM_ExamCardInfo);

        examcard_info = fread(fid);
        str_examcard_info = strcat(char(examcard_info'));

        str_examcard_info_split = strsplit(str_examcard_info,strings_info);

        str_SliceDimensionXInmm = str_examcard_info_split{2};
        str_SliceDimensionXInmm = strsplit(str_SliceDimensionXInmm,',');
        str_SliceDimensionXInmm = str_SliceDimensionXInmm{1};
        SliceDimensionXInmm = str2double(str_SliceDimensionXInmm);

        str_SliceDimensionYInmm = str_examcard_info_split{3};
        str_SliceDimensionYInmm = strsplit(str_SliceDimensionYInmm,',');
        str_SliceDimensionYInmm = str_SliceDimensionYInmm{1};
        SliceDimensionYInmm = str2double(str_SliceDimensionYInmm);

        str_SliceDimensionZInmm = str_examcard_info_split{4};
        str_SliceDimensionZInmm = strsplit(str_SliceDimensionZInmm,',');
        str_SliceDimensionZInmm = str_SliceDimensionZInmm{1};
        SliceDimensionZInmm = str2double(str_SliceDimensionZInmm);

        str_Rows = str_examcard_info_split{6};
        str_Rows = strsplit(str_Rows,',');
        str_Rows = str_Rows{1};
        Rows = str2double(str_Rows);

        str_Columns = str_examcard_info_split{7};
        str_Columns = strsplit(str_Columns,'\n');
        str_Columns = str_Columns{1};
        Columns = str2double(str_Columns);

        fclose(fid);
    else
        fprintf('Mootion Monitoring ExamCards file does not exist
for %s.',FractionFolder)

        SliceDimensionXInmm = nan;
```

```
        SliceDimensionYInmm = nan;
        Rows = nan;
        Columns = nan;
    end

else
    fprintf('ExamCards folder does not exist for %s.',FractionFolder)

    SliceDimensionXInmm = nan;
    SliceDimensionYInmm = nan;
    Rows = nan;
    Columns = nan;
end
```

Function script 2

```
function mm_image = GetMotionMonitoringImage(filepath_mmimage, dir_root)

%strsplit_filepath = strsplit(filepath_mmimage,'\');

[~, ~, ~, Rows, Columns] = GetMotionMonitoringInfo(dir_root);

fid_mmimage = fopen(filepath_mmimage,'r');

% header length: 4084
% total length: 458955
% data_ = fread(fid_mmimage,458955-12500,'*ubit8');
% data = fread(fid_mmimage,500,'*ubit8');

data_header = fread(fid_mmimage,4084,'*ubit8');
%str_data_header = strcat(char(data_header'));

data_image = fread(fid_mmimage,Rows*Columns,'*ubit16');
data_image = data_image';

mm_image = reshape(data_image,[Rows Columns]);
mm_image = circshift(mm_image,[-22 0]);

fclose(fid_mmimage);
```
